# Sweet Saliva Trial: Exploratory Evaluation of Salivary Microbiome Responses to Three Thai Desserts

**DOI:** 10.3390/life16060972

**Published:** 2026-06-09

**Authors:** Sayamon Senaprom, Nuttaphat Namjud, Thunnicha Ondee, Akkarach Bumrungpert, Krit Pongpirul

**Affiliations:** 1 Institute of Nutrition, Mahidol University, Nakhon Pathom 73170, Thailand; sayamon.sen@mahidol.ac.th; 2 College of Allied Health Sciences, Suan Sunandha Rajabhat University, Samut Songkhram Campus, Bangkok 10300, Thailand; nattaphat.na@ssru.ac.th; 3 Center of Excellence in Preventive and Integrative Medicine (CE-PIM), Faculty of Medicine, Chulalongkorn University, Bangkok 10330, Thailand; thunnichaon@yahoo.com; 4 Thai Red Cross Society, Bangkok 10330, Thailand; 5 College of Integrative Medicine, Dhurakij Pundit University, Bangkok 10210, Thailand; abnutrition@yahoo.com; 6 Clinical Research Center, Bumrungrad International Hospital, Bangkok 10110, Thailand; 7 Department of Infection Biology & Microbiomes, Faculty of Health and Life Sciences, University of Liverpool, Liverpool L69 7ZX, UK

**Keywords:** salivary microbiome, Thai desserts, glycemic index

## Abstract

Several studies on diet and microbiome have predominantly focused on the gut microbiome. However, much of the salivary microbiome remains unexplored. This study examined the influence of Thai desserts with varying glycemic indices on the salivary microbiome. A total of 30 healthy adults aged 18–45 years were enrolled in a randomized trial and allocated to one of three intervention groups, with each receiving a culturally specific Thai dessert standardardized to provide 50 g of available carbohydrates: Phetchaburi’s Custard Cake (PCC; low-GI, 192 g), Saraburi’s Curry Puff (SCP; medium-GI, 98 g), or Lampang’s Crispy Rice Cracker (LCRC; high-GI, 68 g). Salivary and dessert microbiome compositions were characterized at baseline and 24 h post-intervention using 16S rRNA sequencing to evaluate bacterial diversity and relative abundance across multiple taxonomic levels. *Firmicutes* were highly abundant (over 76%) in all Thai desserts tested. *Proteobacteria* were found in both SCP (15.1 ± 6.6%) and LCRC (6.5 ± 3.4%). *Actinobacteriota* was slightly higher in the high-GI group (6.3 ± 3.1%) compared to the medium-GI group (3.0 ± 2.2%). *Bacillus* was dominant in PCC, while *Streptococcus*, *Carnobacterium*, and *Clostridium sensu stricto 1* were prevalent in SCP. *Anoxybacillus*, *Clostridium sensu stricto 12*, *Terrilactibacillus, Geobacillus*, and *Weissella* were found in LCRC. Desserts with different types of glycemic index showed modest short-term changes in the relative abundance of some salivary bacteria. Notably, *Porphyromonas* showed a relative increase in the high-GI group compared to the low-GI group (1.8 [0.3, 4.0] vs. −1.9 [−3.2, 0.8], *p* < 0.05), while *Streptococcus*, saccharolytic bacteria, slightly increased in both the high-GI and medium-GI groups. *Prevotella* showed a slight relative decrease in the low-GI group. Although these microorganisms have been previously associated with dysbiosis and periodontitis in longer-term studies, the clinical relevance of these short-term compositional changes remains unclear and should be interpreted with caution. These preliminary findings suggest that local Thai desserts with varying GIs may be associated with transient shifts in salivary microbiota composition; however, whether such changes contribute to dysbiosis or adverse oral health outcomes requires further investigation through longitudinal studies with larger sample sizes.

## 1. Introduction

The human microbiome encompasses diverse microbial communities, with the oral microbiome being the second-most diverse ecosystem [1]. The oral microbiome—also referred to as the oral microbiota or microflora—comprises a wide array of microorganisms inhabiting various niches within the oral cavity, including the gingiva, tongue, palate, lips, and cheeks, and teeth [2,3]. These distinct anatomical surfaces exhibit unique physical properties and salivary exposure levels, which shape site-specific microbial communities [4].

The oral microbiota includes bacteria, archaea, viruses, and protozoa, with an estimated 700 to 1000 microbial species present in the human oral cavity [3,5,6]. A healthy individual’s microbiome typically contains 100 to 200 bacterial species [7]. Bacteria—particularly *Firmicutes*, *Bacillus* spp., *Proteobacteria*, and *Actinomycetes* spp.—dominate the oral microbial community [8]. Nevertheless, each oral niche supports a distinct set of microbes: facultative anaerobes such as *Streptococcus* and *Actinomyces* species are prevalent in saliva, while strict anaerobic like *Bacteroidaceae* and spirochaetes are more common in subgingival areas [6].

Beyond local effects, the oral microbiota contributes to both oral and systemic health [3,9], with mounting evidence linking it to metabolic diseases such as type 2 diabetes [10] and obesity [11]. The oral microbiome is shaped by multiple factors—including dietary habits, oral hygiene, medication use, systemic health, host genetics, and even maternal transmission—making it a dynamic and evolving microbial ecosystem throughout life [9]. Dysregulation of this microbial balance, or oral dysbiosis, has been implicated in various conditions, including dental carries and periodontal diseases.

Key pathogenic contributors to these diseases include *Streptococcus mutans*, *Porphyromonas gingivalis*, *Tannerella forsythia*, and *Aggregatibacter actinomycetemcomitans* [12,13]. In the case of dental caries specifically, *Prevotella* spp., *Lactobacillus* spp., *Dialister* spp., and *Filifactor* spp. have been frequently implicated [3].

Diet is a key environmental factor that influences the oral microbiome by providing nutrients that selectively promote the growth of organisms best adapted to use dietary and host-derived substrates [14]. Major dietary transitions throughout human evolution—such as the shift from hunter–gatherer to agricultural and industrialized diets—have coincided with significant changes in oral microbial composition [15,16]. Interestingly, emerging evidence suggests a bidirectional relationship: not only does diet shape the oral microbiota, but the oral microbiota may also influence host dietary preferences. Specific taxa, such as *Clostridia* and *Prevotella* species, have been linked to taste perception thresholds for sweet, sour, salty, and bitter stimuli, potentially modulating host nutritional behavior in ways that favor microbial survival [14,17,18,19,20].

Oral microbial diversity is often reduced in carious lesions, where the acidic microenvironment selects for acidogenic and aciduric species. For example, the salivary microbiome in individuals with dental caries typically shows elevated levels of *Streptococcus acidophilus* and other acid-tolerant bacteria. During fasting states, bacteria rely on glycoproteins found in saliva and gingival crevicular fluid for nutrition, breaking them down into sugars and amino acids, which are subsequently metabolized into acidic or basic end-products. These are usually neutralized by host buffering systems, maintaining oral pH homeostasis [21]. However, the intake of fermentable carbohydrates, especially sugars and starches, results in acid production that exceeds the buffering capacity, leading to enamel demineralization.

The rate and extent of pH drop are influenced by the carbohydrate types. Simple sugars such as glucose and fructose enter the glycolytic pathway rapidly, causing a swift and pronounced decrease in plague pH and increasing cariogenic potential [22]. In contrast, complex carbohydrates like starch are less readily fermented. Notably, lactose—a disaccharide found in milk—has been shown to be less acidogenic than glucose and fructose in both human and animal studies [23,24].

Diet-induced shifts in oral microbial ecology have also been observed across populations with differing dietary patterns. Comparative studies among hunter–gatherers, traditional agriculturalists, vegetarians, and individuals consuming a Western diet reveal significant variation in core oral microbial species. For example, *Neisseria* and *Haemophilus* are differentially abundant among these groups, with traditional farmers occupying an intermediate microbiome profile between hunter–gatherers and Westerners. Meat-rich diets in hunter–gatherers have been linked to a higher risk of oral disease, whereas vegetarians exhibit altered profiles in both commensals and pathogens [16].

Despite growing interest in diet–microbiota interactions, most research has focused on the gut microbiome, whereas the salivary microbiome remains comparatively underexplored. The oral cavity is continuously exposed to dietary components and hosts a complex microbial ecosystem that may respond rapidly to changes in dietary intake. Given its role as the first site of host–diet–microbe interaction and its potential relevance to both oral and systemic health, understanding how dietary exposures influence the salivary microbiome is of increasing interest. Therefore, this study aimed to investigate the short-term effects of consuming Thai desserts with varying glycemic and nutritional characteristics on the salivary microbiome of healthy adults. This work addresses a novel and culturally relevant intersection between diet and oral microbiota and may contribute to future research on diet–microbiome interactions in Asian populations.

## 2. Materials and Methods

### 2.1. Subjects

Participants in this study were drawn from a larger cohort enrolled in the ‘Glycemic Index and Glycemic Load of Local Famous Desserts in Thailand Project’ [25]. A total of 96 healthy men and women were initially screened for eligibility based on a comprehensive set of inclusion and exclusion criteria. Eligible individuals were required to be between 18 and 45 years of age, with a body mass index (BMI) ranging from 18.5 to 22.9 kg/m^2^. Waist circumference (WC) had to be ≤90 cm for men and ≤80 cm for women. Additional physiological parameters included a systolic blood pressure (SBP) of no more than 120 mmHg, diastolic blood pressure (DBP) no greater than 80 mmHg, and a resting heart rate between 60 and 100 beats per minute.

Biochemical eligibility was established through fasting laboratory tests. Participants were required to have fasting blood sugar (FBS) levels below 100 mg/dL and hemoglobin A1c (HbA1c) between 4.2 and 6.0%. Lipid profiles had to meet the following thresholds: total cholesterol (TC) under 200 mg/dL, triglycerides (TG) below 150 mg/dL, high-density lipoprotein cholesterol (HDL-C) above 40 mg/dL for women or above 50 mg/dL for men, and low-density lipoprotein cholesterol (LDL-C) below 130 mg/dL. Renal and liver function were also considered, with acceptable blood urea nitrogen (BUN) levels ranging from 10 to 20 mg/dL, creatinine (Cr) between 0.6 and 1.2 mg/dL, alanine aminotransferase (ALT) from 0 to 48 IU/L, and aspartate aminotransferase (AST) below 35 IU/L.

To minimize potential confounding factors affecting the oral microbiome, individuals were excluded if they had taken antibiotics, probiotics, or prebiotics within one month prior to screening. Additional exclusion criteria included current smoking or cessation within the past three months, any alcohol consumption, history of gastrointestinal disease, or an episode of diarrhea within one week before the start of the study.

After applying these criteria, 36 individuals were identified as eligible and enrolled into the study. Written informed consent was obtained from all participants before study commencement. Ethical approval for the study protocol was granted by the Institutional Review Board of the Faculty of Medicine, Chulalongkorn University (IRB No. 0215/66; COA No. 0811/2023). All research procedures were conducted in accordance with the ethical principles of the Declaration of Helsinki and in compliance with Good Clinical Practice (GCP) guidelines. The trial was prospectively registered in the Thai Clinical Trials Registry prior to the enrollment of the first participant (TCTR20201008003). Study recruitment and intervention were conducted over a three-month period between October and December 2023.

### 2.2. Study Design

This study was conducted as an open-label, parallel-group, randomized clinical trial aimed at evaluating the impact of Thai desserts with varying glycemic indices (GIs) on the salivary microbiome. As this was an open-label trial, neither allocation concealment nor blinding was implemented. The primary outcome was the change in salivary microbiome composition, assessed by 16S rRNA gene sequencing at baseline and 24 h post-intervention. Secondary outcomes included changes in alpha diversity, beta diversity, and relative abundance of specific bacterial taxa. All procedures took place at the Chulalongkorn International Clinical Research Center in Bangkok, Thailand.

Before the screening visit, participants were reached by telephone and asked to observe a minimum fast of eight hours prior to attending at the study site. On arrival, eligibility was evaluated by trained research staff using a structured screening questionnaire. Body weight, height, and waist circumference (WC) were measured, as well as vital signs including blood pressure and pulse rate. Venous blood sample (10–15 cc) were obtained for biochemical analysis, and participants completed a demographic questionnaire covering self-reported medical history, smoking and alcohol consumption, and physical activity levels.

Anthropometric measurements were obtained in the morning in a fasted state, with participants wearing minimal clothing and no footwear. Height was measured to the nearest 0.1 cm and body weight to the nearest 0.1 kg. BMI (kg/m^2^) was derived by weight divide by height squared (kg/m^2^). WC was assessed at the midpoint between the lower margin of the last palpable rib and the top of the iliac crest. Blood pressure and pulse were assessed with the participant in a seated position using a calibrated instrument. The entire screening procedure required approximately 15–30 min.

The random allocation sequence was generated, participant enrollment was conducted, and group assignment was performed by the research team. Eligible participants were randomly assigned to one of three intervention arms, each corresponding to a dessert group classified by glycemic index, namely low-GI, medium-GI, or high-GI, in a 1:1:1 allocation ratio. Randomization was performed using a simple randomization method with a computer-generated random number sequence. Randomization occurred immediately after screening confirmation.

At the second visit, all enrolled participants were given comprehensive guidance on study procedures and preparation. Participants were advised to avoid desserts, soft drinks, alcohol, yogurt, and fermented foods (e.g., kimchi, pickles, miso, and kombucha), along with oligosaccharide-rich foods (e.g., onions, leeks, garlic, asparagus, Jerusalem artichokes, and chicory root), and any prebiotic or probiotic supplements during the three days prior to the intervention. In addition, participants were instructed to avoid physical exercise throughout the study period to reduce potential confounding effects on the oral microbiome.

Each participant was provided with a food diary to record their dietary intake over the three days prior to the trial. Instructions were given on how to accurately complete the diary, which was estimated to take 5–10 min per day. Participants were also trained to self-collect saliva samples at home and were instructed to fast overnight (8–12 h) before returning for the trial visit. Desserts were consumed in the morning following an overnight fast of 8–12 h. This approach minimized the influence of preceding meals on baseline salivary microbiome composition and allowed for a more controlled assessment of the immediate microbiome response to each dessert.

On the third visit (trial day), participants underwent baseline anthropometric and vital sign measurements, conducted using the same protocol as during screening. They submitted their completed food diaries and saliva samples. The time of saliva sample collection was documented by research staff.

Following these procedures, participants consumed the assigned test dessert, along with 150 mL of still water, with a 15 min period. Each dessert portion was standardized to contain exactly 50 g of available carbohydrates, calculated using the following formula: Serving size (g) = (50 × 100)/carbohydrate content per 100 g.

The carbohydrate content value was derived from prior validated data on Thai desserts, as reported by Namjud et al. (2024) [25]. All dessert samples were sourced from a single production batch to ensure consistency in composition.

**Figure 1:**
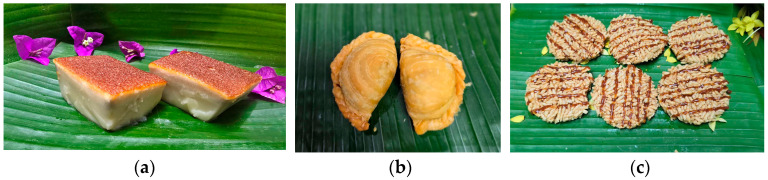
Thai desserts used as test interventions in the randomized trial. (**a**) Phetchaburi’s Custard Cake (PCC)—low glycemic index (GI ≤ 55); (**b**) Saraburi’s Curry Puff (SCP)—medium glycemic index (GI 56–69); and (**c**) Lampang’s Crispy Rice Cracker (LCRC)—high glycemic index (GI ≥ 70). All dessert portions were standardized to provide 50 g of available carbohydrates.

The classification of glycemic index (GI) followed standard definitions: Low GI: ≤55, Medium GI: 56–69, and High GI: ≥70; glucose was used as the reference food (GI = 100) [26]. Although the desserts varied in GI, all of them had a glycemic load (GL) greater than 20 for the experimental portion, placing them in the high-GL category. High-GL foods (GL ≥ 20) are known to provoke greater postprandial glucose and insulin responses per serving than low-GL foods (GL ≤ 10) [27]. The specific Thai desserts used in the intervention are illustrated in Figure 1.

### 2.3. Intervention and Post-Trial Procedures

Participants were randomly allocated to one of three intervention groups, each receiving a standardized portion of a Thai dessert adjusted to provide 50 g of available carbohydrates.

Group 1 (Low GI)

Participants in this group consumed 192 g of Phetchaburi’s Custard Cake (PCC; GI = 53 ± 14), also known as Khanom Maw Kaeng (Figure 1a) [25]. The dessert’s primary ingredients were approximately 29% egg whites, 27% coconut milk, 22% sugar (either palm sugar or refined sugar), and 22% taro. A single serving provided 375 kcal, consisting of 50 g of carbohydrate (including 40.2 g of sugar: sucrose 38.2 g, glucose 0.9 g, fructose 0.5 g, maltose 0.6 g, and lactose not detected), 11 g of protein, and 15 g of fat [28].

Group 2 (Medium GI)

Participants received 98 g of Saraburi’s Curry Puff (SCP; GI = 62 ± 15) or Karipap, a crisp, deep-fried pastry (Figure 1b) [25]. The main ingredients were approximately 41% wheat flour, 22% chicken, 15% potato, 9% onion, and 14% sugar. One serving provided 369 kcal, with 50 g of carbohydrate (including 15.5 g of sugars: sucrose 13.5 g, glucose 0.4 g, fructose 0.4 g, maltose 1.2 g, lactose not detected), 8 g of protein, and 15 g of fat [28].

Group 3 (High GI)

Participants consumed 69 g of Lampang’s Crispy Rice Cracker (LCRC; GI = 149 ± 25), also known as Nang Led or Khao Taen (Figure 1c) [25]. The cracker was made from approximately 59% sticky rice, 26% cane sugar, and 15% watermelon. A single serving provided 308 kcal, with 50 g of carbohydrate (including 15.4 g of sugars: sucrose 10.9 g, glucose 2.4 g, fructose 2.4 g, with maltose and lactose not detected), 3 g of protein, and 11 g of fat [28].

Immediately following dessert consumption, all participants received a standardized breakfast consisting of 180 g of white rice, 180 g of Japanese grilled mackerel (Saba Shioyaki), 30 g of omelet, 25 g of crab stick, and 10 g of sauce. This meal provided approximately 539 kcal, comprising 71 g of carbohydrate, 20 g of protein, and 20 g of fat.

Standardized dietary instructions were given for lunch and dinner on the trial day. Participants were also provided with detailed instructions and materials for saliva sample collection at 24 h post-dessert consumption. They were reminded to avoid physical exercise during the study period to minimize potential confounding effects on the oral microbiome.

At the final visit (24 h post-intervention), participants returned to the study center to submit their saliva samples, with collection times recorded by the research team. Additionally, each participant submitted a completed one-day food record documenting dietary intake following the intervention.

### 2.4. Anthropometrics and Vital Signs Assessment

Anthropometric and physiological measurements were performed under standardized conditions to ensure accuracy and reproducibility. Measurements were obtained in the early morning, with participants fasting, wearing minimal clothing, and no footwear. Height was measured to the nearest 0.1 cm and body weight to the nearest 0.1 kg, using a digital scale with stadiometer (T-Scale M301, T-Scale International Co., Ltd., Taipei, Taiwan), from which body mass index was computed as weight (kg) divided by height squared (m^2^). Waist circumference was assessed using a non-elastic flexible tape at the anatomical midpoint between the lower margin of the last palpable rib and the top of the iliac crest. Vital signs were recorded following a brief resting period. Blood pressure (systolic and diastolic) and pulse rate were obtained with participants seated, using a calibrated automatic sphygmomanometer (Terumo Corporation, Tokyo, Japan).

### 2.5. Dietary Intake Assessment

Dietary intake was evaluated using self-report food records. Prior to the intervention, participants completed a three-day food record documenting all foods and beverages consumed. In addition, a one-day food record was collected on the trial day to capture dietary intake during the intervention period. These records were used using to assess both energy intake and macronutrient composition. Nutrient values were analyzed using INMUCAL nutrient analysis software (Version 4.0) (Institute of Nutrition, Mahidol University, Thailand [29], which is a validated tool for Thai food composition.

### 2.6. Blood Sample Collection and Analysis

At the screening visit, fasting venous blood samples were obtained following an overnight fast of 8–12 h. Different collection tubes were used depending on the assay. Samples for FBS were collected in sodium fluoride (NaF) tubes, while those for HbA1c were drawn into tubes containing ethylenediaminetetraacetic acid (EDTA). Blood intended for lipid profile analyses—including total cholesterol (TC), direct low-density lipoprotein cholesterol (LDL-C), high-density lipoprotein cholesterol (HDL-C), and triglycerides (TG)—as well as liver function tests (AST and ALT), and renal function parameters (creatinine and blood urea nitrogen BUN) was collected in clot-activator tubes.

All samples were centrifuged at 3000 rpm for 20 min at 4 °C, after which plasma and serum were aliquoted and stored at −80 °C until analysis. Biochemical assays were performed at the HIV-NAT AIDS Research Center Laboratory, Faculty of Medicine, Chulalongkorn University, using standardized procedures and quality-controlled platforms.

### 2.7. Saliva Sample Collection

Following the consumption of the assigned test desserts, participants were instructed to refrain from brushing their teeth that evening. Instead, they rinsed their mouths with 50 mL of 0.9% sodium chloride solution before bedtime to ensure gentle cleaning without altering the oral microbiome. On the following morning, immediately upon waking up, participants collected unstimulated saliva samples that had accumulated overnight. To minimize the contamination or alteration of microbial composition, participants were instructed not to brush their teeth, use mouthwash, or drink water prior to collection. The duration of saliva collection was recorded by each participant as a proxy measure of salivary output, as salivary flow rate and volume were not formally measured. These procedures ensured overnight fasting conditions at the post-intervention timepoint, consistent with the 8–12 h overnight fast applied at baseline (Section 2.2).

Saliva was collected into 2 mL Eppendorf tubes prefilled with PrimeStore^®^ Molecular Transport Medium (MTM), a stabilizing solution designed to preserve both DNA and RNA. Samples were kept on ice during transport and transferred to the laboratory, where they were stored at −80 °C within 2 h of arrival. All samples remained frozen until subsequent microbiome analysis.

### 2.8. Thai Dessert Sample Collection

To assess the microbial communities inherent to the intervention foods, three independent samples of each Thai dessert were collected from the same production batch used during the trial. These samples were subjected to metagenomic profiling using 16S rRNA gene sequencing in order to characterize the bacterial community composition associated with the dessert themselves. This parallel analysis was performed to determine whether the test foods harbored distinct microbial signatures that might contribute to observed changes in the salivary microbiome.

### 2.9. DNA Extraction from Saliva and Thai Dessert Samples

Metagenomic DNA was extracted from both saliva and Thai dessert sample using the QIAamp DNA Mini Kit (Qiagen, Hilden, Germany), in accordance with the manufacturer’s protocol. For saliva, 200 µL of sample was processed per extraction, while 0.25 g of each dessert sample was used for parallel extraction. The concentration and purity of all DNA samples were evaluated with a DeNovix QFX fluorometer to ensure suitability for downstream sequencing analyses.

### 2.10. 16S rRNA Gene Next-Generation Sequencing

The V3–V4 hypervariable region of the bacterial 16S rRNA gene was amplified using the Qiagen QIAseq 16S/ITS Region Panel (Qiagen, Hilden, Germany). Sample-specific sequencing adaptors were incorporated into the amplicons through the QIAseq 16S/ITS Region Panel Sample Index PCR Reaction. The resulting DNA libraries, with an average fragment size of approximately 630 base pairs, were assessed for fragment size distribution and integrity using the QIAxcel Advanced System (Qiagen, Hilden, Germany). Library centration were then quantified using a DeNovix QFX Fluorometer (DeNovix Inc., Wilmington, DE, USA) to ensure quality prior sequencing.

Sequencing was performed on the Illumina MiSeq 600 platform (Illumina, San Diego, CA, USA), generating paired-end reads suitable for high-resolution downstream microbiome analysis.

### 2.11. Bioinformatic Analysis

Sequence reads were demultiplexed using 5′ barcode sequences and processed through the DADA2 v1.16.0 pipeline (https://benjjneb.github.io/dada2/, accessed on 1 October 2023). This workflow was employed to identify amplicon sequence variants (ASVs), enabling high-resolution identification of microbial diversity and community [30]. ASV taxonomy was assigned by querying the SILVA reference database (version 138) [31].

Alpha diversity (within-sample diversity) was estimated using Chao1 richness, Shannon diversity, and Faith’s phylogenetic diversity (PD) [32]. Beta diversity (between-sample dissimilarity) was examined through multiple distance metrics, with non-metric multidimensional scaling (NMDS) with Bray–Curtis dissimilarity, and principal coordinates analysis (PCoA) based on weighted UniFrac, unweighted UniFrac, and generalized UniFrac (GUniFrac) distances. Ordination plots were generated using the Phyloseq package [33].

Taxonomic composition was profiled at multiple taxonomic ranks (phylum, class, order, family, and genus) using the ASV annotations. Changes in relative abundance were calculated for each participant and taxon as Relative abundance (%) at 24 h post-intervention − Relative abundance (%) at baseline.

To identify taxa significantly enriched across intervention groups, Linear Discriminant Analysis Effect Size (LEfSe) was employed [34]. LEfSe determined differentially abundant taxa and ranked them according to log_10_-transformed linear discriminant analysis (LDA) scores. Results were visualized using bar plots (LDA scores of discriminative taxa) and cladograms, in which node colors and shading highlighted taxa significantly enriched in specific groups. These analyses supported the detection of potential microbial biomarkers associated with dessert consumption.

### 2.12. Statistical Analysis

Details of sample size calculation are provided in Supplementary Material S1. All analyses were conducted on a per-protocol basis, including only participants who completed the study protocol (*n* = 10 per group). Participant characteristics were summarized using descriptive statistics. Categorical variables were expressed as frequencies and percentages, with between-group comparisons conducted using Pearson’s chi-square test or Fisher’s exact test as appropriate. Continuous variables were expressed as mean ± standard deviation (SD) when normally distributed, or as median and interquartile range (IQR, 25th–75th percentile) when not normally distributed. Distributional assumptions were assessed using the Shapiro–Wilk test and histogram visualization.

Regarding microbiome data, taxon relative abundance spanning phylum through genus levels, along with alpha diversity indices (observed ASVs, Chao1, Shannon, and Faith’s phylogenetic diversity) were compared across groups. One-way ANOVA with Bonferroni’s post hoc test was applied when normality assumptions were satisfied, while the Kruskal–Wallis test followed by Dunn’s multiple comparison test were used for non-normally distributed data. To control for type I error arising from multiple testing, *p*-values were adjusted through the Benjamini–Hochberg false discovery rate (FDR) procedure, with an adjusted *p* < 0.05. All statistical analyses were two-sided, and significance was considered at *p* < 0.05. All statistical analyses were two-sided, and considered significant at *p* < 0.05. Statistical computations were performed using Stata (version 19, STATA Corp., College Station, TX, USA).

Furthermore, the Kruskal–Wallis test was applied as part of the LefSe workflow to detect bacterial biomarkers that were differentially abundant among groups. A threshold LDA score ≥ 1.0 was used to determine the effect size of each significantly enriched taxon. To assess group-level differences in microbial community structure, Permutational Multivariate Analysis of Variance (PERMANOVA) was conducted based on multiple beta diversity metrics, including weighted and unweighted UniFrac PcoA, GuniFrac, and NMDS based on Bray–Curtis dissimilarity. The adonis function within the vegan package in R was used to perform PERMANOVA, with statistical significance set at *p* < 0.05.

## 3. Results

### 3.1. Characteristics of Subjects

**Figure 2:**
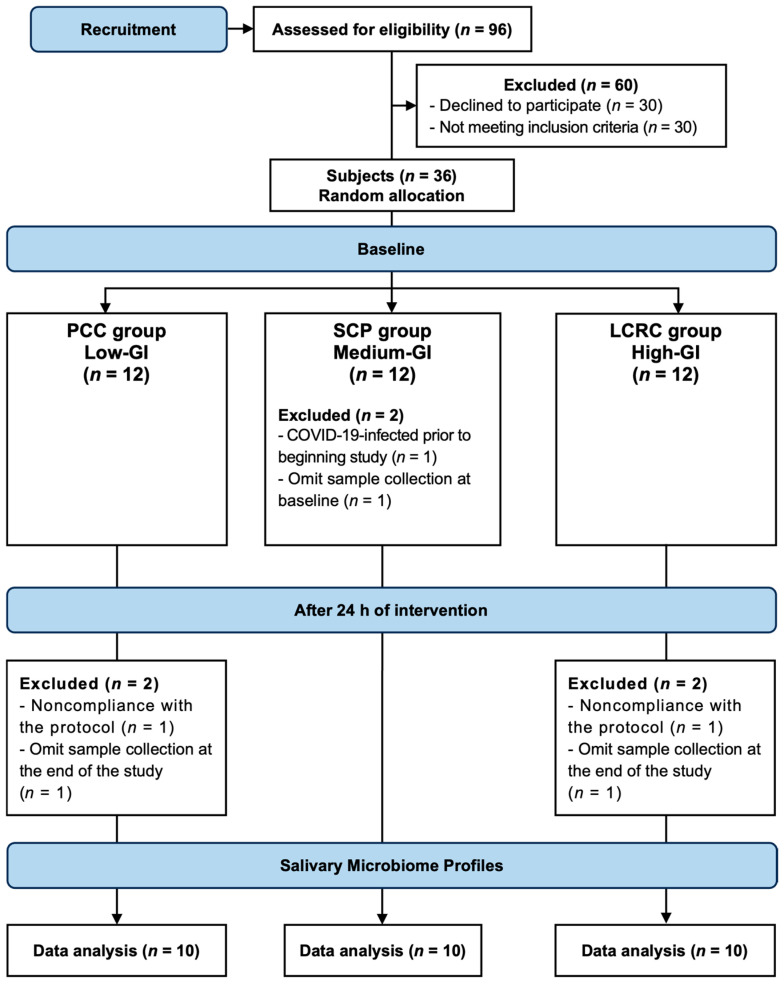
CONSORT flow diagram participant enrollment, allocation, follow-up, and analysis in this randomized clinical trial. A total of 36 subjects was enrolled and randomized to one of three intervention groups: Phetchaburi’s Custard Cake (PCC; low GI), Saraburi’s Curry Puff (SCP; medium GI), and Lampang’s Crispy Rice Cracker (LCRC; high GI). After exclusions due to COVID-19 infection, protocol noncompliance, or omission of saliva collection, 30 participants completed the study and were included in the final salivary microbiome analysis. Each dessert portion contained 50 g of available carbohydrates.

This study followed the Consolidated Standards of Reporting Trials (CONSORT) reporting framework (Figure 2; Supplementary Figure S1). Of 36 healthy volunteers initially enrolled, one participant was excluded due to a COVID-19 infection prior to the intervention. Two additional participants were removed for protocol deviations, including the consumption of dietary fiber products during the run-in period. Three participants were excluded due to non-compliant saliva collection—primarily brushing their teeth shortly before sample collection—which risked compromising microbial integrity.

A total of 30 participants (83%) completed the study and were included in the final analysis, with 10 subjects assigned to each intervention group: Phetchaburi’s Custard Cake (PCC; low GI), Saraburi’s Curry Puff (SCP; medium GI), and Lampang’s Crispy Rice Cracker (LCRC; high GI). Baseline characteristics were comparable across the three groups. No adverse events were reported during the study period.

Across groups, most participants were female (80% in PCC, 80% in SCP, and 90% in LCRC) and predominantly resided in Bangkok. Mean ages were similar—29.2 ± 7.3 years in PCC, 30.7 ± 7.8 years in SCP, and 28.2 ± 7.2 years in LCRC. Anthropometric profiles were with in normal ranges, with mean BMI values of 20.8 ± 1.4, 20.9 ± 1.0, and 20.3 ± 1.5 kg/m^2^, respectively. Waist circumferences were also comparable. Vital signs—including systolic and diastolic blood pressure and pulse—did not differ significantly among groups. All biochemical parameters, including fasting blood sugar, HbA1c, lipid profiles, liver enzymes, BUN, and creatinine, fell within healthy reference ranges and did not differ between groups.

Participants collected follow-up saliva samples approximately 23 h after the intervention, with no significant difference in collection timing across groups. Overall, there were no statistically significant differences in demographic, anthropometric, lifestyle, vital sign, or biochemical characteristics across the three intervention groups (Table 1).

### 3.2. Dietary Consumption

At baseline, two food categories showed statistically significant differences among the three dessert groups. Consumption of bread and baked goods (*p* = 0.038), with no participants in the LCRC group reporting intake, compared to 50% in the PCC group and 40% in the SCP group. Similarly, consumption of eggs and egg products (*p* = 0.037) was reported by 60% of PCC participants, 20% of SCP participants, and 80% of LCRC participants. After the intervention, no significant differences in these food categories were observed among groups (Table 2).

Across all other food groups—including rice, noodles, grains, starchy roots and tubers, legumes/nuts/seeds, vegetables, fruits, meat and poultry, fish and shellfish, processed meats, milk and dairy products, snacks, mixed dishes, soy-based foods, soft drinks, and added sugar—no statistically significant differences were observed among the PCC, SCP, and LCRC groups at baseline or after the intervention (Table 2). Detailed frequency and percentage distributions of food intake during the three days prior to the study are provided in Supplementary Table S1.

There were also no significant differences among groups in total energy intake or overall dietary composition, including macronutrients, dietary fiber, and the proportion of energy derived from carbohydrates, protein, and fat, either at baseline or after the 24 h intervention. However, total sugar intake increased significantly in all groups during the intervention, driven by the consumption of the assigned desserts. The low-GI PCC group exhibited the highest sugar intake (55.6 ± 11.5 g/day), compared with 35.6 ± 21.6 g/day in the SCP group and 36.9 ± 25.7 g/day in the LCRC group. Full details of dietary energy, macronutrient intake, and fiber consumption are available in Supplementary Table S2.

### 3.3. Dessert Microbiome Profiles

**Figure 3:**
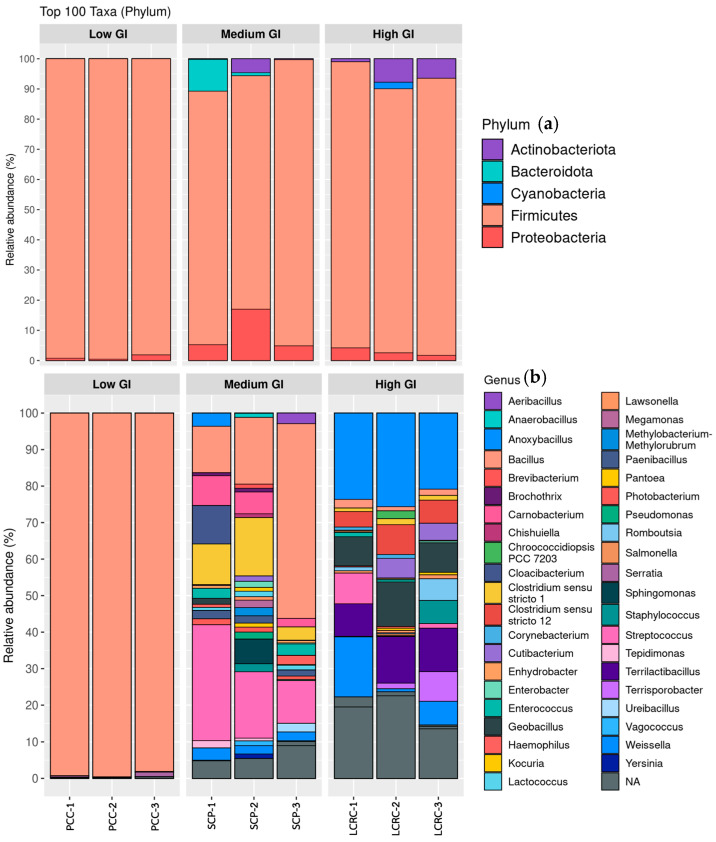
Relative abundance of bacterial phyla and genera among the top 100 identified taxa detected in the Thai desserts—Phetchaburi’s Custard Cake (PCC), Saraburi’s Curry Puff (SCP), and Lampang’s Crispy Rice Cracker (LCRC). Stacked-bar plots illustrate the compositional profiles at the phylum (**a**) and genus (**b**) levels for each dessert type. Detailed relative abundance values for all identified taxa are provided in Supplementary Tables S3 and S4.

Metagenomic profiling revealed distinct microbial compositions across the three Thai desserts. At the phylum level, *Firmicutes* overwhelmingly dominated PCC (low GI), accounting for 98.0 ± 1.6% of the total bacterial abundance, with other phyla detected only in trace amounts. In contrast, *Proteobacteria* were more prominent in SCP (medium GI) and LCRC (high GI), contributing 15.1 ± 6.6% and 6.5 ± 3.4%, respectively. The phylum *Actinobacteriota* was also detected at slightly higher levels in the high GI dessert (6.3 ± 3.1%) compared with the medium-GI dessert (3.0 ± 2.2%) (Figure 3; Supplementary Table S3).

At the genus level (Figure 3), microbial diversity was lowest in PCC, where *Bacillus* constituted 98.3 ± 1.5% of all bacteria, indicating a near-monoculture dominated by this genus. In contrast, both SCP and LCRC exhibited far greater taxonomic heterogeneity (Figure 3; Supplementary Table S4). In SCP, prominent genera included *Streptococcus*, *Carnobacterium*, *Clostridium sensu stricto 1*, and *Bacillus*, representing a mixed community of *Firmicutes* and *Proteobacteria*. LCRC, by comparison, harbored several distinct heat-tolerant and environmental genera such as *Anoxybacillus*, *Clostridium sensu stricto 12*, *Terrilactibacillus*, *Geobacillus*, and *Weissella*, consistent with the high-temperature processing characteristics of LCRC.

Figure 3 provides a stacked-bar representation of the top 100 most abundant taxa at both the phylum and genus levels for all dessert types.

**Figure 4:**
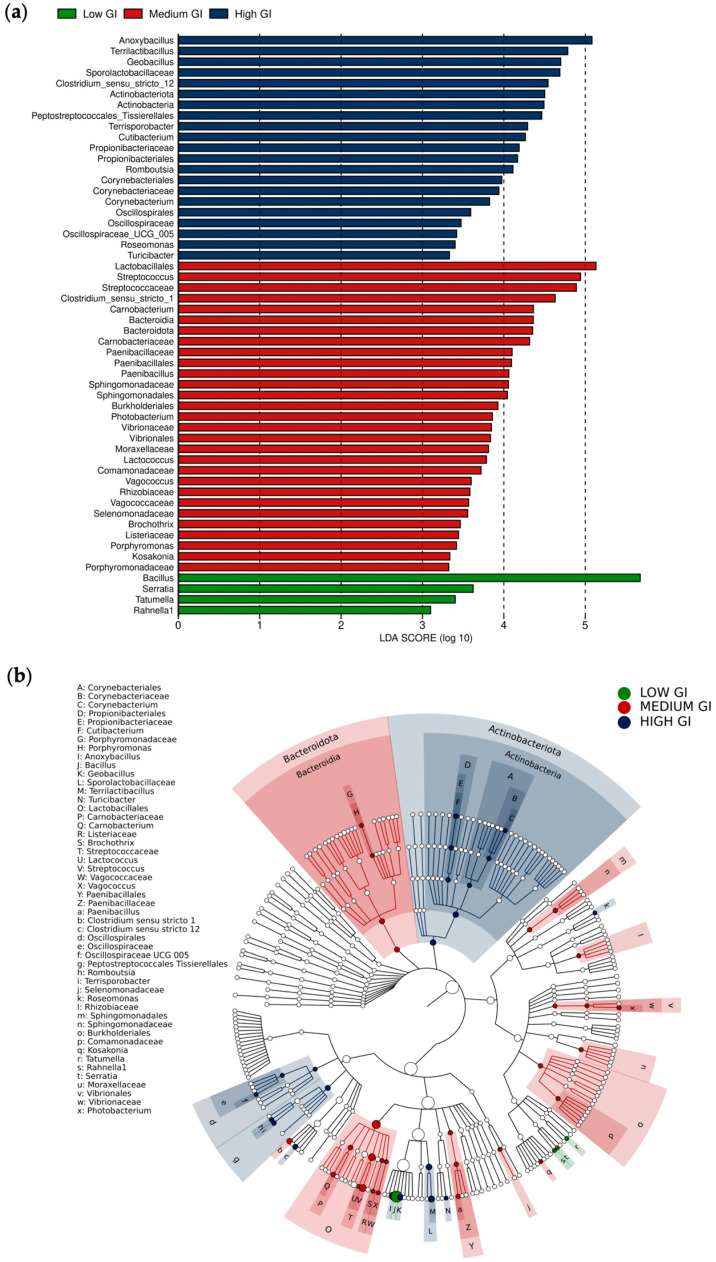
Differentially abundant microbial taxa identified by LEfSe across the Thai dessert microbiomes. (**a**) Bar plot of taxa with significant linear discriminant analysis (LDA) scores, illustrating group-specific microbial biomarkers among the low-GI Phetchaburi’s Custard Cake (PCC), medium-GI Saraburi’s Curry Puff (SCP), and high-GI Lampang’s Crispy Rice Cracker (LCRC). (**b**) Cladogram depicting the phylogenetic relationships and hierarchical distribution of taxa enriched in each dessert group, highlighting the distinct microbial signatures associated with different glycemic index categories.

To identify discriminative microbial signatures, LEfSe analysis was performed (Figure 4). Several genera exhibited significantly elevated LDA scores (>3), indicating strong group-specific enrichment:

- Low GI (PCC): The core biomarkers included *Bacillus*, *Tatumella*, *Serratia*, and *Rahnella1* (Proteobacteria); all significantly enriched (*p* < 0.05).
- Medium GI (SCP): A broader and more diverse set of taxa was enriched, including *Bacteroidota*, *Lactobacillales*, *Sphingomonadaceae*, *Porphyromonas*, *Carnobacterium*, *Brochothrix*, *Lactococcus*, *Streptococcus*, *Vagococcus*, *Paenibacillus*, *Clostridium sensu stricto 1*, *Photobacterium*, and *Kosakonia* (*p* < 0.05).
- High GI (LCRC): Biomarkers were dominated by rare environmental or thermophilic genera, including *Actinobacteriota*, *Corynebacterium*, *Cutibacterium*, *Anoxybacillus*, *Geobacillus*, *Terrilactibacillus*, *Turicibacter*, *Romboutsia*, *Clostridium sensu stricto 12*, *Oscillospiraceae UCG 005*, *Terrisporobacter*, and *Roseomonas* (*p* < 0.05).

### 3.4. Impact of Thai Desserts on the Salivary Microbiome

**Figure 5:**
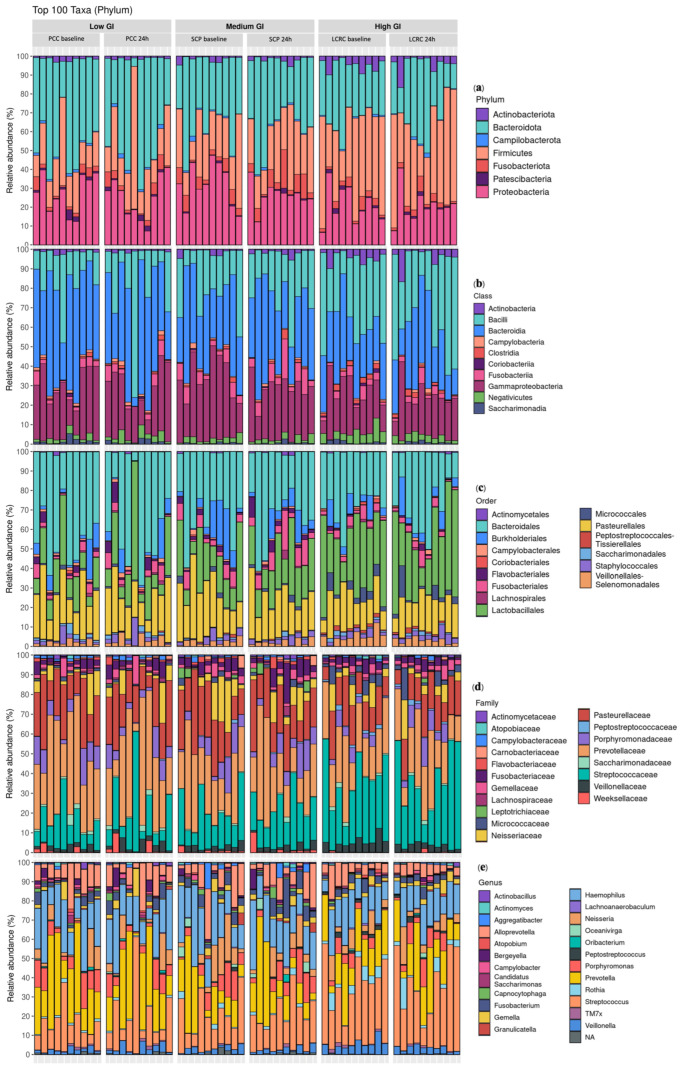
Relative abundance of salivary microbiota at the (**a**) phylum, (**b**) class, (**c**) order, (**d**) family, and (**e**) genus levels among the top 100 most abundant taxa. Stacked-bar plots illustrate the taxonomic composition of the salivary microbiome across hierarchical levels using only the top 100 identified taxa. Complete relative abundance profiles for all detected phyla, classes, orders, families, and genera are provided in Supplementary Tables S5–S9.

The taxonomic identification of bacteria through 16S rRNA gene sequencing indicated that the salivary microbiota in subjects consisted of 12 phyla, 17 classes, 49 orders, 90 families, and 177 genera (Supplementary Tables S5–S9). In the analysis of the top 100 taxa, the salivary microbiome profile was predominantly represented by seven phyla, 10 classes, 15 orders, 19 families, and 24 genera, as illustrated in Figure 5.

Across all samples, a total of 12 phyla were detected: *Actinobacteriota*, *Bacteroidota*, *Campilobacterota*, *Cyanobacteria*, *Desulfobacterota*, *Firmicutes*, *Fusobacteriota*, *Gemmatimonadota*, *Patescibacteria*, *Proteobacteria*, *Spirochaetota*, and *Synergistota*. Eight phyla were enriched among the top 100 taxa. At the phylum level, *Bacteroidota* was the most dominant (37.6 ± 14.0%), followed by *Firmicutes* (28.0 ± 14.4%), *Proteobacteria* (23.3 ± 8.7%), *Fusobacteriota* (4.8 ± 3.5%), *Actinobacteriota* (3.9 ± 3.2%), *Patescibacteria* (0.98 ± 0.92%), *Campilobacterota* (0.79 ± 0.53%), and *Spirochaetota* (0.60 ± 0.96%).

Mild, group-specific shifts were observed after dessert consumption. In the PCC (low-GI) group, *Firmicutes* increased slightly at 24 h (24.4 ± 19.2%) relative to baseline (18.5 ± 10.1%), while *Bacteroidota* decreased modestly (44.0 ± 18.8% vs. 46.9 ± 12.3%). In the SCP (medium-GI) group, *Fusobacteriota* increased at 24 h (8.0 ± 5.6% vs. 5.8 ± 2.8%), whereas *Proteobacteria* decreased (23.6 ± 6.0% vs. 27.6 ± 9.1%). In the LCRC (high-GI) group, *Actinobacteriota* decreased slightly over 24 h (5.8 ± 4.1% vs. 8.1 ± 3.7%) (Supplementary Table S5). Although modest, these changes suggest dessert-specific micro-shifts in salivary composition within one day.

At the class level, *Bacteroidia* was consistently the most abundant class across all dessert groups, ranging from 28% to 47%, followed by *Bacilli* (15–32%) and *Gammaproteobacteria* (20–28%) (Supplementary Table S6). At the order level, the community was dominated by *Bacteroidales* (27–44%), *Lactobacillales* (12–29%), *Pasteurellales* (14–19%), and *Fusobacteriales* (3–8%) (Supplementary Table S7).

At the family level, *Prevotellaceae* was the most abundant family (22–36%), followed by *Streptococcaceae* (12–28%), *Pasteurellaceae* (14–19%), *Neisseriaceae* (4–8%), and *Porphyromonadaceae* (3–8%) (Supplementary Table S8).

With over 177 genera detected (Supplementary Table S9), genus-level profiling showed that *Streptococcus* (12–29%), *Prevotella* (16–24%), *Haemophilus* (14–18%), and *Alloprevotella* (6–12%) were the dominant genera across participants. Other frequently observed genera included *Porphyromonas* (4–8%), *Neisseria* (4–7%), *Fusobacterium* (3–6%), *Veillonella* (2–5%), *Actinomyces* (1–4%), *Rothia* (1–4%), *Gemella* (2–3%), *Capnocytophaga* (1–3%), *Aggregatibacter* (1–2%), and low-abundance genera such as *Campylobacter* and *Leptotrichia* (both ~1%).

Overall, these findings indicate that while the core salivary microbiome remained largely stable, consumption of Thai desserts introduced subtle but detectable shifts in the relative abundance of several bacterial groups over a 24 h period.

### 3.5. Changes in the Relative Abundance of Salivary Microbiota After 24 h of Thai Dessert Consumption

**Figure 6:**
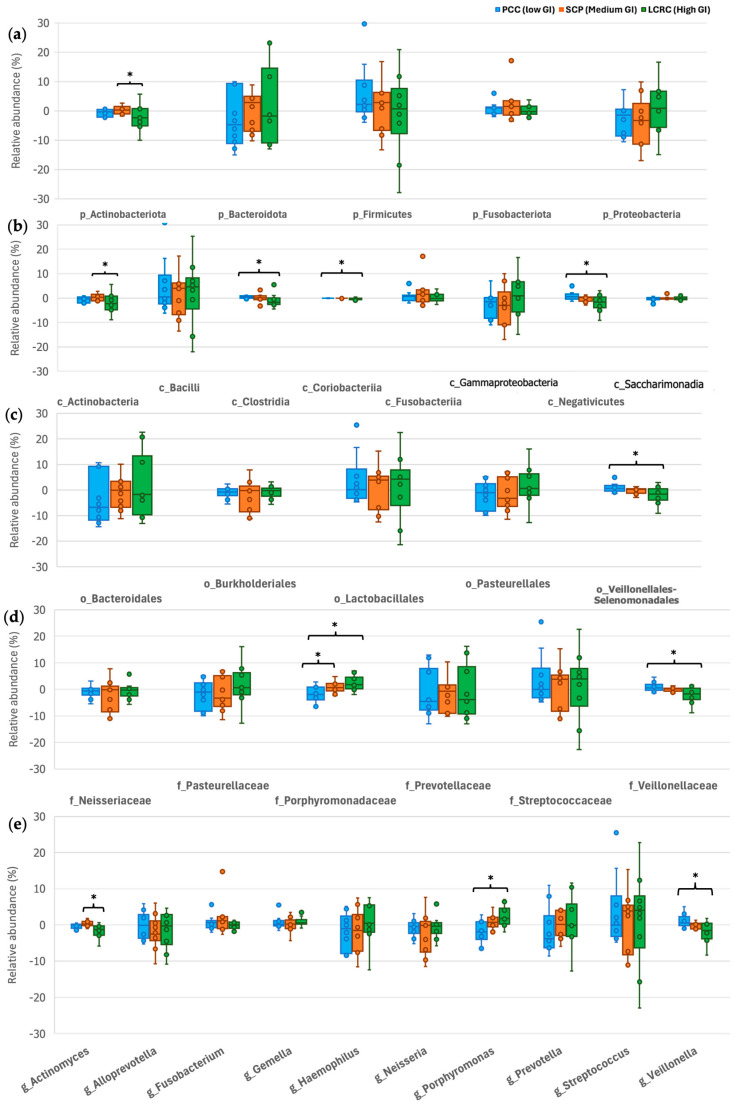
Boxplots showing changes in the relative abundance of salivary microbiota 24 h after ingestion of Thai desserts with different glycemic indices. Comparative shifts in microbial abundance are displayed at the (**a**) phylum, (**b**) class, (**c**) order, (**d**) family, and (**e**) genus levels for the PCC (low GI), SCP (medium GI), and LCRC (high GI) groups. Statistical analyses were performed using the Kruskal–Wallis test, followed by Dunn’s multiple comparison tests with Benjamini–Hochberg correction (adjusted *p* < 0.05). Asterisks (*) denote statistically significant differences between groups. Detailed quantitative results are provided in Supplementary Tables S10–S14.

Figure 6 illustrates the shifts in salivary microbial composition across phylum, class, order, family, and genus levels following 24 h ingestion of Thai desserts with different glycemic indices, with detailed quantitative values provided in Supplementary Tables S10–S14.

At the phylum level (Figure 6a; Supplementary Table S10), the median relative abundance of *Firmicutes* showed a slight increase in the low-GI (PCC) group, whereas *Bacteroidota* exhibited a modest decrease compared with the SCP and LCRC groups. *Actinobacteriota* decreased significantly in the high-GI (LCRC) group relative to the SCP group, while *Proteobacteria* showed a slight increase in the LCRC group compared with both PCC and SCP.

At the class level (Figure 6b; Supplementary Table S11), *Actinobacteria*, Coriobacteriia, and Negativicutes were significantly reduced in the LCRC group compared with the SCP and PCC groups, respectively. Fusobacteriia declined slightly in the LCRC group, whereas Gammaproteobacteria increased slightly relative to the PCC and SCP groups. Bacilli showed a small increase in both the LCRC and SCP groups compared with PCC.

At the order level (Figure 6c; Supplementary Table S12), Veillonellales–Selenomonadales decreased significantly in the LCRC group compared with PCC. *Lactobacillales* and *Pasteurellales* displayed slight increases in the LCRC group relative to PCC and SCP, respectively. In contrast, Bacteroidales showed a modest decrease in the PCC group compared with SCP and LCRC.

At the family level (Figure 6d; Supplementary Table S13), Veillonellaceae decreased significantly in the LCRC group, while Porphyromonadaceae decreased significantly in PCC. Pasteurellaceae showed a slight decrease in SCP, whereas Streptococcaceae increased slightly in both the SCP and LCRC groups.

At the genus level (Figure 6e; Supplementary Table S14), the high-GI (LCRC) group exhibited an increase in Porphyromonas, accompanied by decreases in *Actinomyces* and *Veillonella*. *Streptococcus* increased slightly in both the LCRC and SCP groups, while *Alloprevotella* decreased slightly in SCP. In the PCC group, *Prevotella* showed a modest decline relative to both SCP and LCRC.

Taken together, these findings suggest that although most changes were modest, different glycemic index dessert types induced distinct taxon-specific responses in the salivary microbiome within 24 h.

### 3.6. Salivary Microbial Biomarkers Associated with Thai Dessert Consumption Identified by LEfSe Analysis

**Figure 7:**
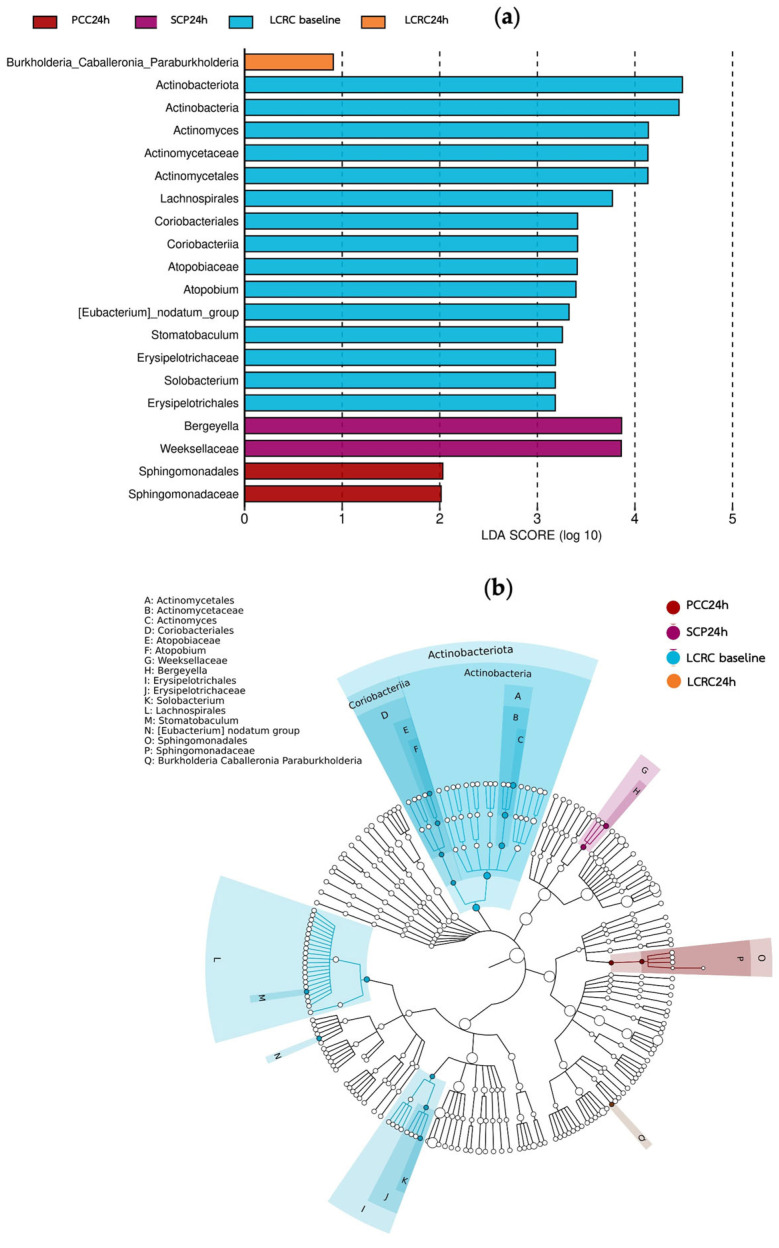
Differentially abundant salivary microbial taxa identified by LEfSe across dessert groups at baseline and 24 h post-consumption. (**a**) Bar plot showing taxa with significant linear discriminant analysis (LDA) scores, highlighting microbial biomarkers associated with the PCC (low-GI), SCP (medium-GI), and LCRC (high-GI) groups at baseline and after 24 h. (**b**) Cladogram illustrating the phylogenetic relationships and hierarchical structure of enriched taxa, demonstrating distinct shifts in salivary microbial patterns between baseline and post-intervention within each dessert group: PCC (low-GI), SCP (medium-GI), and LCRC (high-GI) at baseline and after 24 h.

LEfSe analysis revealed distinct salivary microbial biomarkers associated with the consumption of Thai desserts with different glycemic indices (Figure 7). At baseline, the high-GI (LCRC) group exhibited the enrichment of several taxa belonging to *Actinobacteriota*, including *Actinobacteriota*, *Actinomycetaceae*, *Actinomyces,* Atopobiaceae, *Atopobium*, and members of the Eubacterium nodatum group, along with Erysipelotrichaceae, *Solobacterium*, and *Stomatobaculum* (*p* < 0.05).

Following the 24 h intervention, additional biomarkers emerged. In the high-GI group, genera belonging to the *Proteobacteria* cluster—*Burkholderia*, *Caballeronia*, and *Paraburkholderia*—were identified as core enriched taxa (*p* < 0.05). In the medium-GI (SCP) group, *Weeksellaceae* and *Bergeyella* showed significant enrichment after dessert intake (*p* < 0.05). The low-GI (PCC) group demonstrated enrichment of *Sphingomonadales* and *Sphingomonadaceae*, both within the phylum *Proteobacteria*, at 24 h (*p* < 0.05).

Collectively, these results indicate that even within a short timeframe, consumption of Thai desserts with differing glycemic indices produces distinct taxon-specific shifts in salivary microbial biomarkers, reflecting both baseline microbial community differences and food-driven responses.

### 3.7. Alpha Diversity of Salivary Microbiome Profiles

High-quality 16S rRNA gene sequencing generated a total of 8,644,181 paired-end reads, corresponding to an average sequencing coverage of 70.6% across the 30 participants. The average number of reads per sample at baseline was 165,251.9 ± 21,786.9 (PCC), 154,735.8 ± 18,364.4 (SCP), and 109,126.9 ± 16,264.7 (LCRC), and at 24 h post-intervention, it was 166,238.7 ± 34,021.5 (PCC), 153,076.6 ± 19,079.7 (SCP), and 115,988.2 ± 17,793.5 (LCRC) (Supplementary Table S15). Alpha diversity metrics—including observed ASVs, Chao1 richness, Shannon diversity, and Faith’s phylogenetic diversity (PD whole tree)—were used to evaluate within-sample microbial diversity across the three dessert groups.

At baseline, significant differences were observed between the high-GI (LCRC) and medium-GI (SCP) groups in observed ASVs, Chao1 richness, and PD whole tree, indicating variation in richness and phylogenetic breadth prior to the intervention. In contrast, Shannon diversity, reflecting both richness and evenness, showed no significant differences among groups at baseline.

**Figure 8:**
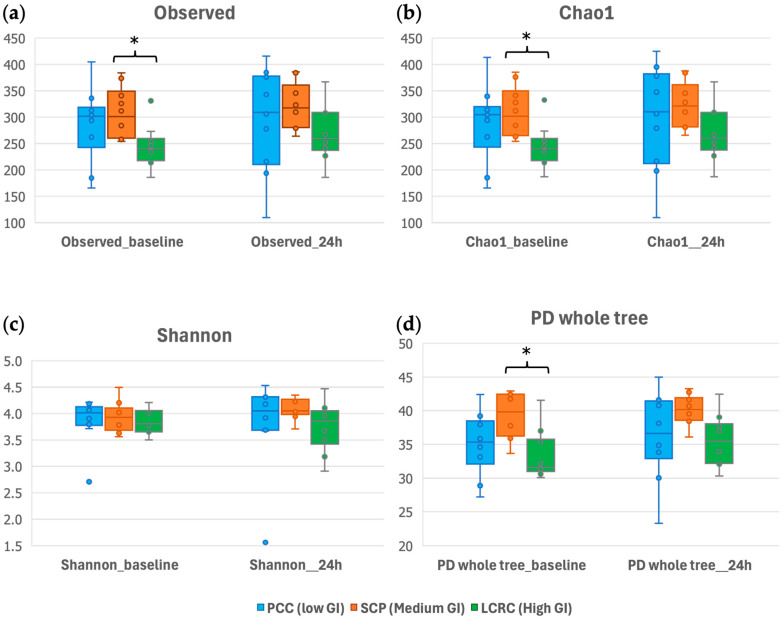
Alpha diversity indices of the salivary microbiota before and after Thai dessert consumption across glycemic index groups. Boxplots display changes in (**a**) observed ASVs, (**b**) Chao1 richness estimator, (**c**) Shannon diversity index, and (**d**) Faith’s phylogenetic diversity (PD whole tree) for the PCC (low GI), SCP (medium GI), and LCRC (high GI) groups at baseline and 24 h after ingestion. Statistical comparisons were performed using one-way ANOVA or the Kruskal–Wallis test, followed by Dunn’s post hoc tests with Benjamini–Hochberg correction (adjusted *p* < 0.05). * *p* < 0.05, indicates statistically significant difference between groups. Detailed alpha diversity values are provided in Supplementary Table S16.

After the 24 h intervention, no significant differences in any alpha diversity index were detected among the PCC, SCP, and LCRC groups (Figure 8; Supplementary Table S16). These findings suggest that although baseline diversity varied between groups, consumption of Thai desserts with different glycemic indices did not induce measurable changes in overall salivary microbial richness or diversity within 24 h.

### 3.8. Beta Diversity of Salivary Microbiome Profiles

**Figure 9:**
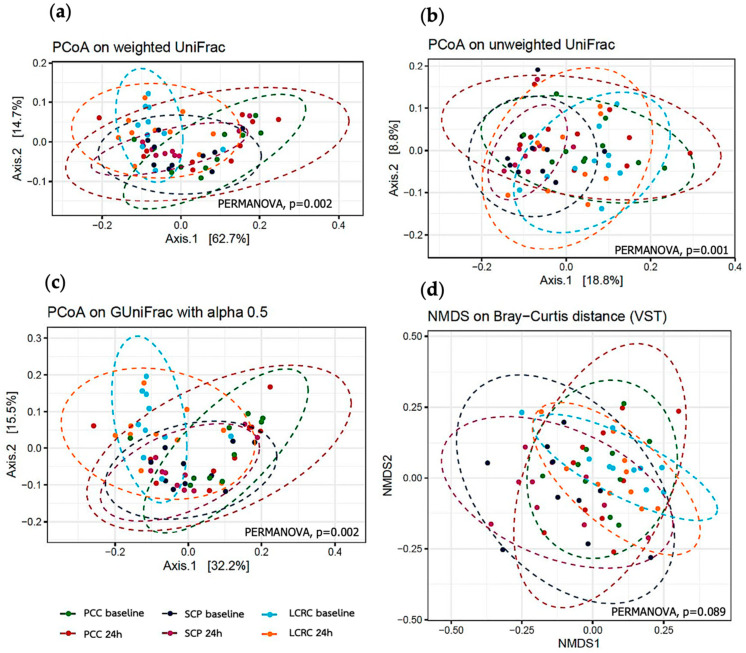
Beta diversity analysis of the salivary microbiota before and after consumption of Thai desserts with different glycemic indices. Two-dimensional ordination plots illustrate differences in microbial community composition among the PCC (low GI), SCP (medium GI), and LCRC (high GI) groups at baseline and 24 h post-intervention. Principal coordinates analysis (PCoA) was performed using (**a**) weighted UniFrac, (**b**) unweighted UniFrac, and (**c**) generalized UniFrac (GUniFrac, α = 0.5) distance metrics. (**d**) Non-metric multidimensional scaling (NMDS) was conducted based on Bray–Curtis dissimilarity. Significant clustering between groups was detected for several distance metrics (*p* < 0.05, PERMANOVA), indicating overall community structure differences across groups, which may reflect both baseline heterogeneity and post-intervention shifts.

Beta diversity analyses demonstrated that the overall salivary microbial community structure differed across dessert groups at both timepoints combined. PCoA based on weighted UniFrac, unweighted UniFrac, and generalized UniFrac (GUniFrac) distances showed clear separation among groups (Figure 9a–c), with PERMANOVA indicating significant differences (*p* = 0.002, 0.001, and 0.002, respectively). However, as these analyses incorporated both baseline and 24 h samples, the observed between-group differences may partly reflect pre-existing baseline heterogeneity rather than intervention-specific effects exclusively. In contrast, NMDS based on Bray–Curtis dissimilarity revealed no significant clustering (PERMANOVA *p* = 0.089; Figure 9d), suggesting that compositional differences were more apparent when accounting for phylogenetic relationships than when using non-phylogenetic metrics.

Within-group comparisons revealed additional shifts over time. In the PCC (low-GI) group, both unweighted UniFrac and weighted UniFrac distances showed significant differences between baseline and 24 h samples (*p* < 0.001 and *p* = 0.008, respectively), indicating detectable structural changes in the salivary microbiome following consumption of the low-GI dessert. In the LCRC (high-GI) group, a significant baseline-to-24 h difference was observed with weighted UniFrac (*p* = 0.041), suggesting more subtle but measurable community alterations.

Taken together, these findings suggest that the salivary microbiome exhibits mild but distinct structural differences across dessert groups, with within-group analyses indicating shifts within 24 h after consuming Thai desserts, with phylogenetically informed metrics appeared to detect these differences more sensitively than Bray–Curtis dissimilarity.

## 4. Discussion

This study examined how three Thai desserts differing in glycemic index—Phetchaburi’s Custard Cake (PCC; low-GI), Saraburi’s Curry Puff (SCP; medium-GI), and Lampang’s Crispy Rice Cracker (LCRC; high-GI)—influence the salivary microbiome within a 24 h period. Using 16S rRNA gene sequencing, we identified 12 bacterial phyla in the salivary samples, a range consistent with prior oral microbiome studies. These phyla largely overlapped with those cataloged in the Human Oral Microbiome Database, which lists 13 phyla in healthy oral ecosystems [35]. In our dataset, the top 100 taxa included eight enriched phyla, dominated by *Bacteroidota*, *Firmicutes*, and *Proteobacteria*—together accounting for more than 80% of total relative abundance. This distribution aligns with previous studies showing that *Firmicutes*, *Actinobacteria*, *Proteobacteria*, *Fusobacteria*, *Bacteroidetes*, and *Spirochaetes* constitute the vast majority of oral bacteria in healthy individuals [36].

At the genus level, *Streptococcus*, *Prevotella*, *Haemophilus*, and *Alloprevotella* were the most abundant, each exceeding 10% relative abundance. Several genera with relative abundances above 1%, including *Porphyromonas*, *Neisseria*, *Fusobacterium*, *Veillonella*, *Actinomyces*, *Rothia*, *Gemella*, and *Capnocytophaga*, matched those described in established oral microbiome literature [37,38]. Many of these taxa have been described as components of the normal oral microbiota and may contribute to oral ecological balance, metabolic cross-feeding, and the maintenance of mucosal surfaces. Beneficial genera such as *Lactobacillus*, *Bifidobacterium*, *Streptococcus*, and *Weissella*—which have been studied for probiotic roles in oral health—were also detected [39].

### 4.1. Short-Term Responses to Dessert Consumption

A 24 h intervention is a narrow window, but even within this period, modest compositional shifts were observed. Notably, Porphyromonas increased in the high-GI (LCRC) group. This genus includes *Porphyromonas gingivalis*, a keystone periodontal pathogen capable of inducing dysbiosis by altering community interactions rather than dominating through absolute abundance [40,41]. Its proteolytic metabolism supports both its own growth and that of neighboring species [42,43]. Although our study did not directly identify species-level changes, the observed increase in *Porphyromonas* following high-GI dessert intake may be associated with short-term shifts in the oral microbial environment. As this study was exploratory in nature, these findings should be interpreted with caution, and further investigation is needed to clarify whether such changes reflect GI-related dietary effects or other dessert-specific factors.

Carbohydrate excess promotes the growth of saccharolytic and acidogenic bacteria such as *Streptococcus*, *Actinomyces*, and *Veillonella*, and has been associated with enamel demineralization and caries formation in the prior literature [14,44,45,46]. In our data, *Streptococcus* increased slightly in the medium- and high-GI groups, while *Actinomyces* decreased in the high-GI group. The LEfSe analysis supported these patterns: *Streptococcus* was a biomarker in the SCP group, whereas several *Actinobacteriota*-associated genera—*Actinomyces*, *Atopobium*, *Stomatobaculum*, and *Solobacterium*—were biomarkers at baseline in the LCRC group but decreased post-intervention. This shift is consistent with previous findings suggesting that *Actinomyces* are nitrogen-metabolizing bacteria that may contribute to moderating acidification in plaque ecology [47]. However, these observed changes should be interpreted with caution, and further investigation is needed to clarify whether they reflect GI-mediated effects or dessert-specific microbial contributions.

### 4.2. Dietary Context and Nitrate-Reducing

Diet affects oral microbiota beyond carbohydrate metabolism. Nitrate-rich foods, especially leafy vegetables, enhance nitrate-reducing bacteria (e.g., *Rothia*, *Neisseria*) and can modulate oral acidity and nitric oxide production [48,49,50]. Although no significant differences in vegetable consumption were noted between groups, our results showed that *Veillonella* decreased in the high-GI group, and *Prevotella* slightly declined in the low-GI group. *Rothia* and *Neisseria* remained stable. The short intervention period and limited dietary control may have constrained our ability to detect diet–microbiome interactions beyond sugar intake.

### 4.3. Distinct Microbial Biomarkers Across Desserts

LEfSe analysis provided a clearer functional interpretation of dessert-specific microbiota.

- Low-GI (PCC): *Sphingomonadales* and *Sphingomonadaceae* were observed post-intervention.
- Medium-GI (SCP): *Weeksellaceae* and *Bergeyella* became enriched.
- High-GI (LCRC): *Burkholderia*, *Caballeronia*, and *Paraburkholderia* (Proteobacteria) were more prominent after 24 h.

These distinct signatures may indicate that the specific composition of the desserts, including their ingredient profiles, processing methods, and intrinsic microbiota, may shape oral microbial patterns through different metabolic pathways or ecological responses, independent of or in addition to glycemic index effects. However, it is important to note that the present study does not directly demonstrate transfer, persistence, or colonization of dessert-associated taxa within the oral microbiome. The contribution of intrinsic dessert microbiota to the observed salivary microbiome changes therefore remains speculative and should be regarded as a hypothesis to be tested in future studies using appropriate study designs, such as tracking specific dessert-associated taxa longitudinally before and after consumption. The open nature of the oral cavity—continuously exposed to food, drink, air, and environmental microbes—may contribute to the oral microbiota being responsive to short-term perturbations [13]. With saliva containing up to 700 species and more than 500 million bacterial cells per milliliter [3,51], rapid fluctuations in microbiome structure may occur.

### 4.4. Alpha and Beta Diversity Responses

No significant differences in alpha diversity indices were observed between groups after 24 h, suggesting that richness and evenness remained stable despite taxonomic shifts. However, baseline differences in ASVs, Chao1, and phylogenetic diversity between the LCRC and SCP groups reflected natural interindividual variation. These baseline differences indicate that randomization did not fully balance microbiome-related characteristics across groups, and therefore between-group comparisons of post-intervention alpha diversity should be interpreted with caution, as observed differences may partly reflect pre-existing heterogeneity rather than intervention-specific effects.

In contrast, beta diversity revealed subtle but significant structural changes. Weighted and unweighted UniFrac distances showed shifts in the PCC group from baseline to 24 h, indicating changes in both the presence/absence and phylogenetic structure of taxa. The LCRC group also showed a significant change in weighted UniFrac. However, it should be noted that the between-group PERMANOVA analyses incorporated both baseline and 24 h samples; therefore, the significant differences observed may partly reflect baseline heterogeneity rather than intervention-related shifts exclusively. Within-group comparisons, which directly contrasted baseline and 24 h samples within each group, provide a more reliable indicator of intervention-related changes, though causal interpretation remains limited given the open-label design and small sample size. These patterns suggest that oral microbial communities are dynamic and may response to dietary exposures, even within a day. Their nonequilibrium behavior may be influenced by host interactions, pH cycles, microbial cross-feeding, and genetic mechanisms such as horizontal gene transfer [52].

### 4.5. Strengths and Limitations

A major strength of this study is the use of well-defined, culturally relevant Thai desserts with a standardized carbohydrate content of 50 g of available carbohydrates across all groups, allowing a controlled assessment of GI-related effects. The randomized controlled design and high-depth 16S sequencing further strengthened taxonomic resolution and analytical robustness. However, several limitations must be acknowledged. First, the findings of this study reflect short-term associations in relative microbial abundance only and should not be interpreted as evidence of causality, metabolic effects, or clinical oral health outcomes. Second, the short 24 h follow-up period constrains interpretation, as it remains unclear whether the observed microbial shifts are transient or sustained over time. Third, although participants received dietary guidance, limited control over non-breakfast meals means that real-world variability in meal composition may have independently influenced microbiome responses, further precluding causal conclusions. Fourth, although carbohydrate content was standardized at 50 g across all groups, the desserts differed in fat, protein, and primary ingredients. No significant differences in overall dietary composition were observed during the intervention, except for total sugar intake. Nevertheless, microbiome changes cannot be attributed exclusively to glycemic index, and this compositional variability should be considered an important confounding factor. Fifth, salivary flow rate and saliva volume were not formally measured; the duration of saliva collection was recorded as a proxy measure. As salivary flow and hydration status may influence microbial concentration and relative abundance, this represents an additional limitation that should be addressed in future studies. Sixth, although participants were advised to avoid desserts, soft drinks, alcohol, yogurt, and fermented foods during the study period, as described in Section 2.2, this was a recommendation rather than a strict prohibition. Minor dietary deviations were observed in individual participants, and given the small sample size, such deviations may have influenced group-level microbiome analyses. Seventh, baseline differences in alpha diversity indices were observed between groups, suggesting that randomization may not have fully balanced microbiome-related characteristics. As a result, between-group comparisons of post-intervention findings may partly reflect this pre-existing heterogeneity rather than intervention-specific effects, which further limits causal interpretation. This should be considered when interpreting the findings. Eighth, the beta diversity analyses (PERMANOVA) were conducted on data pooling both baseline and 24 h samples across groups. Therefore, the significant between-group differences observed may partly reflect pre-existing baseline heterogeneity rather than intervention-specific effects exclusively, and should be interpreted with caution. Ninth, the homogeneity of multivariate dispersion was not formally assessed using PERMDISP or betadisper. Therefore, the significant PERMANOVA results should be interpreted with caution, as they may partly reflect differences in within-group dispersion rather than differences in group centroids exclusively. Finally, the modest sample size and lack of long-term follow-up limit the generalizability of the findings. Therefore, although a randomized controlled design was employed, this study should be regarded as exploratory in nature, and the results should be interpreted accordingly.

### 4.6. Future Directions

The present study provides preliminary evidence of short-term associations between glycemic index and salivary microbiome composition; however, causal mechanisms remain to be established. Future work should include larger cohorts, longer observation periods, multiple sampling timepoints, and controlled meal plans to determine whether the transient microbial shifts observed here persist or carry clinical relevance. To better isolate the effect of glycemic index from dessert-specific confounders, future studies should also incorporate multiple desserts per GI category with matched nutritional profiles. Species-level identification, functional metagenomics, and metabolomics integration would further clarify the biological and metabolic pathways involved, as these aspects were beyond the scope of the current study.

## 5. Conclusions

This randomized controlled trial provides preliminary evidence that Thai desserts with different glycemic indices may be associated with short-term shifts in the salivary microbiome. Within 24 h, high-GI dessert consumption was associated with an increase in *Porphyromonas*, while *Streptococcus* showed modest increases in both the medium- and high-GI groups. In contrast, *Prevotella* showed a slight relative decline in the low-GI group. Although these changes were subtle, they involve taxa that have been reported in relation to oral ecological balance and, in some cases, oral dysbiosis and periodontal risk.

However, these findings should be interpreted with caution. As the study used a single dessert per GI category, the observed microbiome shifts may reflect dessert-specific effects, including differences in ingredient composition and intrinsic dessert microbiota, rather than glycemic index per se. The results therefore provide exploratory, hypothesis-generating evidence rather than definitive conclusions regarding GI-mediated effects on the oral microbiome. Nonetheless, this study contributes preliminary data on how culturally specific, carbohydrate-rich foods may be associated with short-term shifts in salivary microbiota, supporting a growing body of evidence connecting diet, oral microbiota, and systemic health.

The results also suggest the potential of salivary microbiome profiling as a non-invasive tool for investigating dietary exposures, through its utility as a biomarker for metabolic responses requires further validation. Further studies with longer follow-up, multiple desserts per GI category with matched nutritional profiles, controlled diets, and functional microbiome analysis are needed to clarify whether these short-term microbial shifts carry long-term oral or systemic health implications.

## Figures and Tables

**Table 1:** Baseline characteristics of participants by glycemic index group.

Characteristics		PCC (Low-GI, *n* = 10)		SCP (Medium-GI, *n* = 10)	LCRC (High-GI, *n* = 10)	*p*-Value
Age, years		29.2 ± 7.3; 29.4 (22.7–33.1)		30.7 ± 7.8; 28.2 (25.4–40.0)	28.2 ± 7.2; 26.8 (21.0–34.1)	0.766
Sex, Male/Female		2 (20%)/8 (80%)		2 (20%)/8 (80%)	1 (10%)/9 (90%)	1.000
Residence						
	Bangkok	8 (80%)		6 (60%)	8 (80%)	0.668
	Metropolitan areas	2 (20%)		4 (40%)	2 (20%)	
Physical Activity						
	No	4 (40%)		1 (10%)	2 (20%)	0.224
	Sometimes	5 (50%)		9 (90%)	8 (80%)	
	Usually	1 (10%)		0 (0%)	0 (0%)	
Anthropometrics						
	BMI, kg/m^2^	20.8 ± 1.4; 20.5 (19.7–22.1)		20.9 ± 1.0; 20.9 (19.8–21.9)	20.3 ± 1.5; 20.1 (19.2–22.1)	0.590
	WC, cm	72.2 ± 5.3; 72.5 (70.0–75.0)		73.1 ± 6; 71.5 (68.0–77.0)	69.6 ± 5.7; 69.5 (68.0–71.0)	0.373
Vital signs						
	SBP, mmHg	110.0 ± 10.9; 112.0 (100.0–120.0)		110.9 ± 12.5; 109.5 (101.0–117.0)	107.9 ± 13.7; 110.0 (102.0–116.0)	0.859
	DBP, mmHg	66.1 ± 6.9; 66.0 (61.0–69.0)		65.9 ± 7.5; 63.0 (61.0–67.0)	67.1 ± 10.0; 70.0 (62.0–73.0)	0.942
	Pulse, bmp	78.4 ± 11.8; 79.5 (67.0–89.0)		85.5 ± 13.2; 83.0 (77.0–94.0)	83.6 ± 14.7; 81.0 (73.0–88.0)	0.475
Blood biochemical parameters						
	FBS, mg/dL	83.7 ± 6.6; 82.5 (79.0–86.0)		83.9 ± 5.9; 83.5 (80.0–89.0)	85.6 ± 5.8; 84.0 (81.0–90.0)	0.748
	HbA1c, %	5.0 ± 0.4; 5 (4.8–5.4)		5.0 ± 0.3; 5.0 (4.8–5.2)	5.1 ± 0.3; 5.0 (5.0–5.3)	0.842
	TC, mg/dL	180.6 ± 25.6; 187.5 (150.0–201.0)		169.9 ± 17.8; 173.5 (161.0–180.0)	176.2 ± 25.9; 180.5 (156.0–200.0)	0.596
	TG, mg/dL	61.7 ± 32.7; 48.0 (42.0–63.0)		63.8 ± 19.4; 70.5 (45.0–79.0)	55.3 ± 16.9; 55.5 (41.0–68.0)	0.533
	HDL-C, mg/dL	62.2 ± 10; 60.5 (57.0–69.0)		60.8 ± 14.8; 58.5 (57.0–63.0)	63.5 ± 14.3; 66.0 (49.0–75.0)	0.901
	LDL-C, mg/dL	108.4 ± 33.4; 123.5 (91.0–131.0)		97.7 ± 26.6; 102.0 (81.0–111.0)	106.4 ± 29.0; 107.0 (101.0–128.0)	0.698
	BUN, mg/dL	9.8 ± 1.7; 9.3 (9.0–10.1)		11.5 ± 3.1; 11.0 (9.9–11.9)	11.0 ± 5.7; 9.3 (8.0–10.5)	0.282
	Creatinine, mg/dL	0.7 ± 0.2; 0.7 (0.6–0.8)		0.7 ± 0.2; 0.7 (0.6–0.8)	0.8 ± 0.2; 0.7 (0.6–0.7)	0.557
	ALT, IU/L	15.0 ± 3.7; 15.0 (12.0–17.0)		14.0 ± 3.2; 14.0 (11.0–17.0)	14.6 ± 8.7; 11.5 (10.0–14.0)	0.347
	AST, IU/L	17.4 ± 3.3; 17.5 (16.0–20.0)		18.0 ± 2.1; 18.0 (17.0–18.0)	15.5 ± 4.0; 15.0 (13.0–17.0)	0.209
Duration of saliva collection, h						
		23.0 ± 2.5; 22.3 (21.5–22.5)	23.0 ± 3.1; 21.8 (21.5–22.6)		22.8 ± 1.4; 22.5 (22.3–24.0)	0.597

Data are shown as mean ± SD and median (25th–75th percentile). No significant difference were observed between groups. Fisher’s exact test was applied for categorical variables. One-way ANOVA was used for normally distributed continuous variables, and the Kruskal–Wallis test was used for non-normally distributed continuous variables; normality was assessed using the Shapiro–Wilk test. Physical activity was categorized as follows: sometimes (1–4 times/week) and usually (≥5–6 times/week). Abbreviations: GI, glycemic index; PCC, Phetchaburi’s Custard Cake; SCP, Saraburi’s Curry Puff; LCRC, Lampang’s Crispy Rice Cracker; BMI, body mass index; WC, waist circumference; SBP, systolic blood pressure; DBP, diastolic blood pressure; FBS, fasting blood sugar; HbA1c, hemoglobin A1c; TC, total cholesterol; TG, triglycerides; HDL-C, high-density lipoprotein cholesterol; LDL-C, low-density lipoprotein cholesterol; BUN, blood urea nitrogen; Cr, creatinine; ALT, alanine aminotransferase; AST, aspartate aminotransferase.

**Table 2:** Comparison of food group consumption across Thai dessert groups at baseline and after the 24 h test period.

	Baseline (Pre-Intervention)				After 24 h (Post-Intervention)			
Food Group	PCC (*n* = 10)	SCP (*n* = 10)	LCRC (*n* = 10)	*p*-Value	PCC (*n* = 10)	SCP (*n* = 10)	LCRC (*n* = 10)	*p*-Value
Rice	8 (80)	10 (100)	8 (80)	0.507	10 (100)	10 (100)	10 (100)	-
Bread/bake	5 (50)	4 (40)	0 (0)	0.038	2 (20)	2 (20)	4 (40)	0.668
Noodles	4 (40)	3 (30)	1 (10)	0.450	3 (30)	3 (30)	4 (40)	1.000
Grains and products	2 (20)	1 (10)	2 (20)	1.000	2 (20)	0 (0)	0 (0)	0.31
Starchy roots and tubers	1 (10)	0 (0)	0 (0)	1.000	2 (20)	2 (20)	2 (20)	1.000
Legumes/nuts/seeds	1 (10)	3 (30)	1 (10)	0.574	2 (20)	0 (0)	0 (0)	0.31
Vegetables	6 (60)	7 (70)	8 (80)	0.879	8 (80)	6 (80)	9 (90)	0.430
Fruits	7 (70)	4 (40)	3 (30)	0.272	4 (40)	1 (10)	2 (20)	0.43
Beef and pork	8 (80)	3 (30)	5 (50)	0.106	5 (50)	4 (40)	5 (50)	1.000
Poultry	5 (50)	8 (80)	6 (60)	0.510	6 (60)	5 (50)	6 (60)	1.000
Fish and shellfish	4 (40)	4 (40)	4 (40)	1.000	10 (100)	8 (80)	10 (100)	0.31
Processed meat and fish	4 (40)	4 (40)	3 (30)	1.000	8 (80)	6 (60)	10 (100)	0.122
Egg and egg products	6 (60)	2 (20)	8 (80)	0.037	8 (80)	8 (80)	9 (90)	1.000
Milk and dairy	2 (20)	0 (0)	0 (0)	0.310	1 (10)	3 (30)	0 (0)	0.286
Thai desserts	1 (10)	1 (10)	1 (10)	1.000	10 (100)	10 (100)	10 (100)	-
Ice cream	0 (0)	0 (0)	1 (10)	1.000	0 (0)	0 (0)	0 (0)	-
Snacks	0 (0)	0 (0)	2 (20)	0.310	0 (0)	1 (10)	1 (10)	1.000
Mixed dishes	5 (50)	7 (70)	3 (30)	0.202	5 (50)	2 (20)	3 (30)	0.5
Soybean foods	3 (30)	2 (20)	1 (10)	0.847	2 (20)	2 (20)	2 (20)	1.000
Soft drinks	2 (20)	2 (20)	3 (30)	1.000	3 (30)	0 (0)	3 (30)	0.195
Added sugar	4 (40)	1 (10)	1 (10)	0.301	2 (20)	2 (20)	1 (10)	1.000

Data are presented as n (%). Significant associations between food group consumption and dessert group were evaluated using Fisher’s exact test or Pearson’s chi-square test (*p* < 0.05). Full statistical details are provided in Supplementary Table S2.

## Data Availability

The original contributions presented in this study are included in the article/Supplementary Materials. Further inquiries can be directed to the corresponding author(s).

## Supplementary Materials

The following supporting information can be downloaded at https://www.mdpi.com/article/10.3390/life16060972/s1, Figure S1: Study Design Overview of the Sweet Saliva Trial; Supplementary Material S1: Sample Size Calculation; Table S1: Comparison of food group consumption across Thai dessert groups at baseline and during the test period; Table S2: Comparison of nutrient intakes at baseline and after intervention across dessert groups; Table S3: The relative abundance of identified microbiome at the phylum level in Thai desserts; Table S4: The relative abundance of identified microbiome at the genus level in Thai desserts; Table S5: The relative abundance of identified salivary microbiomes at the phylum level by group; Table S6: The relative abundance of identified salivary microbiomes at the class level; Table S7: The relative abundance of identified salivary microbiomes at the order level; Table S8: The relative abundance of identified salivary microbiomes at the family level; Table S9: The relative abundance of identified salivary microbiomes at the genus level; Table S10: Changes in the relative abundance of salivary microbiomes at the phylum level after 24 h of Thai dessert consumption; Table S11: Changes in the relative abundance of salivary microbiomes at the class level after 24 h of Thai dessert consumption; Table S12: Changes in the relative abundance of salivary microbiomes at the order level after 24 h of Thai dessert consumption; Table S13: Changes in the relative abundance of salivary microbiomes at the family level after 24 h of Thai dessert consumption; Table S14: Changes in the relative abundance of salivary microbiomes at the genus level after 24 h of Thai dessert consumption; Table S15: Quality profiles of bacterial taxa in saliva; Table S16: Alpha diversity of bacterial communities in saliva. Reference [53] is cited in the Supplementary Materials.

## Abbreviations

The following abbreviations are used in this manuscript:

PCC	Phetchaburi’s Custard Cake
SCP	Saraburi’s Curry Puff
LCRC	Lampang’s Crispy Rice Cracker
GIs	Glycemic indices
GL	Glycemic Load
BMI	Body Mass Index
WC	Waist Circumference
SBP	Systolic Blood Pressure
DBP	Diastolic Blood Pressure
FBS	Fasting Blood Sugar
HbA1c	Hemoglobin A1c
TC	Total Cholesterol
TG	Triglycerides
HDL-C	High-Density Lipoprotein Cholesterol
LDL-C	Low-Density Lipoprotein Cholesterol
BUN	Blood Urea Nitrogen
Cr	Creatinine
ALT	Alanine Aminotransferase
AST	Aspartate Aminotransferase
GCP	Good Clinical Practice
NaF	Sodium Fluoride
EDTA	Ethylenediaminetetraacetic Acid
MTM	Molecular Transport Medium
LEfSe	Linear Discriminant Analysis Effect Size
LDA	Linear Discriminant Analysis
SD	Standard Deviation
IQR	Interquartile Range
PERMANOVA	Permutational Multivariate Analysis of Variance
CONSORT	Consolidated Standards of Reporting Trials
