# Supplementary material for: Sweet Saliva Trial: Exploratory Evaluation of Salivary Microbiome Responses to Three Thai Desserts
Source: Life (Basel). 2026 Jun 9;16(6):972. doi: 10.3390/life16060972 (PMC13302562; doi:10.3390/life16060972)
Supplement: Supplementary file 1 [file life-16-00972-s001.zip › Supplementary Material S1.pdf]

Supplementary Material S1

Sample Size Calculation

Sample size was calculated based on the study by Mano et al. (2018), using data on Actinobacteria relative abundance following dietary intervention. A minimum of 9 participants per group was required, and accounting for an estimated 20% dropout rate, the adjusted sample size was 12 participants per group, for a total of 36 participants. This sample size was applied to the salivary microbiome analysis conducted concurrently with a gut microbiome study using the same participants.

Ref: Mano F, Ikeda K, Joo E, Fujita Y, Yamane S, Harada N, et al. The Effect of White Rice and White Bread as Staple Foods on Gut Microbiota and Host Metabolism. Nutrients. 2018;10(9):1323.
