# Supplementary figures and images for: Sweet Saliva Trial: Exploratory Evaluation of Salivary Microbiome Responses to Three Thai Desserts
Source: Life (Basel). 2026 Jun 9;16(6):972. doi: 10.3390/life16060972 (PMC13302562; doi:10.3390/life16060972)

## Supplementary Figure

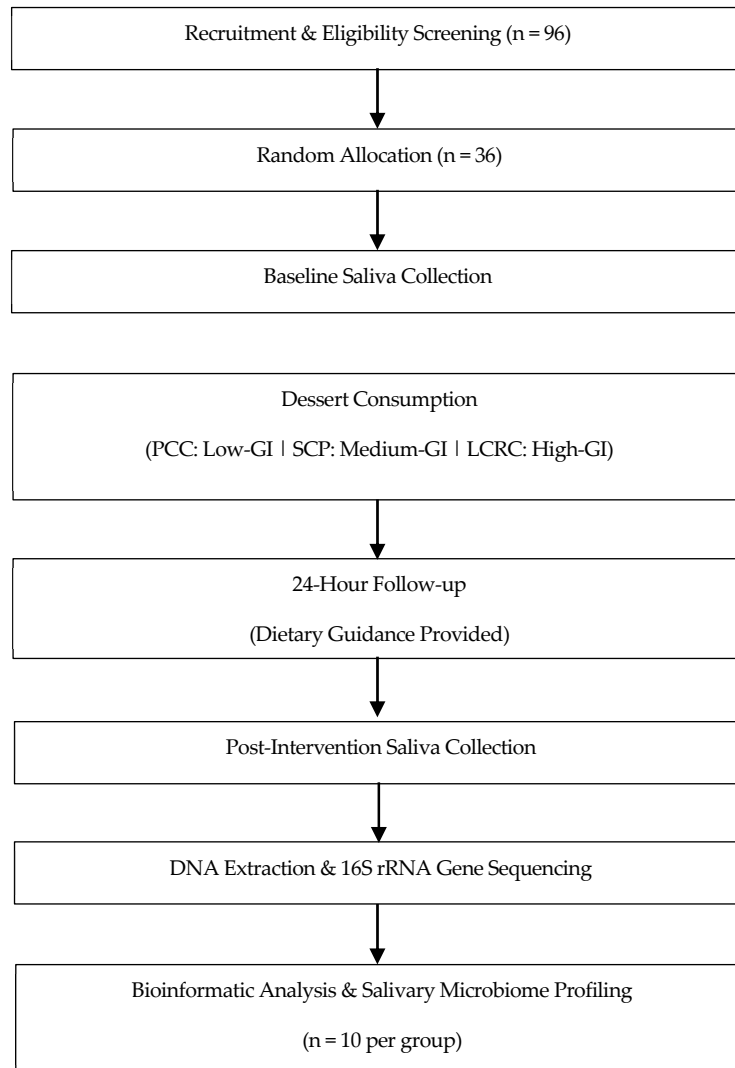

**Figure S1.** Study Design Overview of the Sweet Saliva Trial

Supplement: Supplementary file 1 [file life-16-00972-s001.zip › Supplementary Figure S1.pdf]
